# Effect of Pilates on Pain and Health-Related Quality of Life in Fibromyalgia Patients: A Systematic Review and Meta-Analysis

**DOI:** 10.3390/jcm13237447

**Published:** 2024-12-06

**Authors:** Chalisa Nithuthorn, Natapohn Chaipichit, Thammasorn Jeeraaumponwat, Mart Maiprasert, Piyameth Dilokthornsakul

**Affiliations:** 1 Department of Anti-Aging and Regenerative Medicine College of Integrative Medicine, Dhurakij Pundit University, Laksi, Bangkok 10210, Thailand; 2 Center for Medical and Health Technology Assessment (CM-HTA), Department of Pharmaceutical Care, Faculty of Pharmacy, Chiang Mai University, Chiang Mai 50200, Thailand; natapohn.ch@cmu.ac.th; 3 Department of Social Medicine, Khon Kaen Hospital, Srichan Rd, Nai Mueang, Mueang, Khon Kaen 40000, Thailand; t.jeeraaumponwat@cpird.in.th

**Keywords:** pilates, fibromyalgia, pain, health-related quality of life

## Abstract

**Background:** Pilates is one of the non-pharmacological interventions for fibromyalgia (FM). However, its impacts on pain and health-related quality of life (HRQOL) in fibromyalgia patients (FMPs) are inconclusive. This study aimed to assess the effects of Pilates on pain and HRQOL among FMPs. **Methods:** A systematic review and meta-analysis were conducted. Four databases—PubMed, ScienceDirect, Scopus, and Cochrane CENTRAL—along with one grey literature source, Google Scholar, were searched for randomized controlled trials comparing Pilates with other exercises or usual care in FMPs. The outcomes were pain and HRQOL. A meta-analysis was performed using a random-effects model. **Results:** Six studies were included *(n* = 265). We found that the effects of Pilates from each individual study on pain were inconsistent. Our pooled analysis of visual analog scale (VAS) scores demonstrated significant pain reduction (mean difference (MD), −0.71, 95%CI, −1.33 to −0.10, *p* = 0.023; (*I*^2^ = 29.3%, *p* = 0.226)). However, neither the algometric score (AS) nor tender point count (TPC) showed an insignificant difference (AS: MD, −0.43, 95%CI, −2.60 to 1.74, *p* = 0.700; (*I*^2^ = 0.0%, *p* = 0.654); TPC: MD, −0.16, 95%CI, −2.22 to 1.89, *p* = 0.520; (*I*^2^ = 0.0%, *p* = 0.515)). Regarding HRQOL, Pilates showed statistically significant improvements on the Fibromyalgia Impact Questionnaire (FIQ) (MD, −7.28, 95%CI, (−12.06 to −2.49), *p* = 0.003; (*I*^2^ = 95.7%, *p* < 0.001)). A sensitivity analysis of three RCTs (*n* = 176) based on the ACR 2010 supported this finding (MD, −7.68, 95% CI, −8.60 to −6.76, *p* < 0.001; (*I*^2^ = 0.0%*, p =* 0.832)) with non-important heterogeneity. **Conclusions:** Pilates may benefit FMPs. It could reduce pain and improve HRQOL. Given the small number of studies and the presence of data heterogeneity, future high-quality RCTs would provide a clearer conclusion.

## 1. Introduction

Fibromyalgia is a widespread pain syndrome accompanied by fatigue, depression, anxiety, cognitive impairment, and sleep disturbance. It is more prevalent in women than in men, with a female-to-male ratio of 3:1 [1,2]. The worldwide prevalence of fibromyalgia was estimated to be approximately 2.7% in 2013 [3]. This condition leads to substantial economic burdens, both directly and indirectly [3,4,5]. A study in Quebec, Canada, reported an annual healthcare cost of 4000 Canadian dollar per FMP, which was 30% higher than for those without the condition [4]. Similarly, in Japan, the direct medical cost of fibromyalgia was estimated at JPY 1,943,000 per year, while the indirect economic cost due to work absenteeism reached JPY 2,827,000 annually [5].

The precise pathogenesis of fibromyalgia remains unknown. However, several mechanisms have been hypothesized and demonstrated in some patients, including peripheral and central hyperexcitability at the spinal or brainstem level, altered pain perception, and somatosensory dysfunction [1,2,6]. The treatment for fibromyalgia typically focuses on symptom management. Exercise is a non-pharmacological intervention for the condition [1,2,6]. A recent review indicated that supervised aerobic exercise training and strength training can improve physical capacity and clinical symptoms in FMPs. Pilates is one such exercise modality [7,8].

Pilates is a specific exercise approach founded on the teachings of Joseph Pilates (1880–1967) [9]. It emphasizes core strength, posture, coordination, and flexibility, aiming to improve overall body health. Reported benefits include improvements in pain, fitness, functional ability, flexibility, and balance [9]. As a mind–body integrative exercise, Pilates also helps reduce stress due to the need for focus and breath control during practice [9]. In recent years, the use of exercise-based interventions, including Pilates, for normal population and FMPs have gained increasing attention worldwide [10]. Studies in Turkey and Brazil have demonstrated Pilates’ effectiveness in reducing pain and improving physical function in FMPs [10,11]. However, there is still a lack of evidence of Pilates in other countries which might have different treatment effects. Further studies are needed to assess the feasibility and efficacy of Pilates in different populations.

A distinctive feature of Pilates is its use of specialized equipment, such as the Reformer machine, which utilizes springs to provide resistance [10]. This resistance intensifies exercises, engaging deeper muscle layers, which can contribute to musculoskeletal strengthening and improved posture [9]. Because of its comprehensive benefits, Pilates has been suggested as a potential treatment for fibromyalgia [9,10]. However, its effects on pain management and HRQOL in individuals with fibromyalgia remain inconclusive.

Despite the growing interest in exercise-based interventions for fibromyalgia, there is limited evidence on the effectiveness of Pilates specifically, particularly when compared to other exercise modalities and usual care. While previous studies have explored various exercise forms, such as aerobic and strength training, there remains a need for a more comprehensive understanding of Pilates’ impact on both pain reduction and health-related quality of life in FMPs [8]. This study aims to address this gap by performing a systematic review and meta-analysis of randomized controlled trials (RCTs) to assess the efficacy of Pilates compared to other exercise interventions and standard treatments for fibromyalgia.

## 2. Materials and Methods

### 2.1. Search Strategies

A comprehensive and systematic literature search was conducted adhering to the Preferred Reporting Items for Systematic Reviews and Meta-Analyses (PRISMA) guidelines [12] and the Cochrane Collaboration Handbooks [12,13]. Two review authors (CN and NC) independently searched four databases—PubMed, ScienceDirect, Scopus, and Cochrane CENTRAL—without language restrictions from their inception to 1 February 2024. Search terms were constructed using Boolean operators and MeSH terms based on the following concepts: “Pilates”, “health-related quality of life”, “pain”, and “FIQ”. Search strategies were adapted for each database as needed. A sample PubMed search used in this study was ((((((((pilates) OR (pilates based exercise)) OR (pilates training)) AND (HRQOL)) OR (health related quality of life)) OR (QOL)) OR (quality of life)) AND (pain)) AND (FIQ)). In addition to the systematic database search, we also reviewed the references of the included studies to identify any potentially relevant articles that were not captured in the initial search. Furthermore, we searched gray literature from Google Scholar to ensure a comprehensive search strategy in line with AMSTAR-2 recommendations [14]. The review protocol was registered in PROSPERO (CRD42024517175).

### 2.2. Study Selection

We included studies based on the PICO structure as follows:

Population (P): adults diagnosed with fibromyalgia;

Intervention (I): Pilates interventions;

Comparison (C): usual care or other exercise modalities;

Outcome (O): pain reduction and HRQOL, with the primary outcomes measured by the VAS, AS, TPC for pain, and FIQ for HRQOL.

Exclusion criteria: Studies involving pregnant women or participants with other significant medical conditions were excluded from the review.

Article selection process: Two independent review authors (CN and NC) screened titles, abstracts, and full texts of potential articles. Any disagreements were resolved through consensus with a third review author (PD).

### 2.3. Data Extraction and Quality Assessment

The data on patients and their baseline characteristics, intervention type, length of sessions, frequency, duration of exercise, age, gender, American College of Rheumatology (ACR) criteria used for fibromyalgia diagnosis, FIQ score, and VAS score were all collected. The quality of included studies was assessed independently by two review authors (CN and NC) using the PEDro scale [15,16]. The scale comprised 11 items, of which one eligibility criterium was not scored (related to external validity) in the present study. The remaining 10 items (random allocation, concealed allocation, baseline comparability, blind subjects, blind therapist, blind assessors, measures ≥ 85% of the sample, intention to treat, between-group comparison, point estimates, and availabilities) were scored, with each item worth 1 point for a total possible score of 10 points. Scores were categorized into four levels: poor (0–3 points), fair (4–5 points), good (6–8 points), and excellent (9–10 points). Studies with a score ≥ 6 are considered high quality [15,16]. The high-quality trials enhance the methodological soundness of the analysis, reduce bias, and ensure a more accurate representation of the true effects of the interventions studied [17].

### 2.4. Statistical Analysis

Data from studies reporting outcomes within the same category were pooled to determine the overall effect size and its associated 95% confidence interval (CI). Treatment effects were considered statistically significant when the *p*-value was less than or equal to 0.05 [18]. Mean changes in outcome variables were calculated for each study by subtracting baseline values from post-intervention measures. Pooled standard deviations of these mean changes were subsequently used to calculate overall mean differences (MDs) between the intervention and comparator group. All analyses employed a random-effects model [19]. The use of a random-effects model is appropriate because it improves the interpretation of diverse data, enhances generalizability, and ensures that pooled results more accurately reflect the range of potential real-world scenarios [19]. The Cochran chi-squared test for heterogeneity was performed and considered statistically significant if *p* ≤ 0.10 [20]. Heterogeneity was also quantified with *I*^2^-statistics, whereby 0–40% may not be important, 30–60% may represent moderate heterogeneity, 50–90% may represent high heterogeneity, and 75–100% is defined as considerable heterogeneity [13]. To explore potential sources of heterogeneity, sensitivity and subgroup analyses were conducted. For the sensitivity analysis, a leave-one-out approach was used, and studies utilizing the ACR 2010 diagnostic criteria were grouped. Several subgroup analyses were performed based on types of control, type of Pilates exercise, and patients’ age.

## 3. Results

### 3.1. Search Results and Study Characteristics

**Figure 1:**
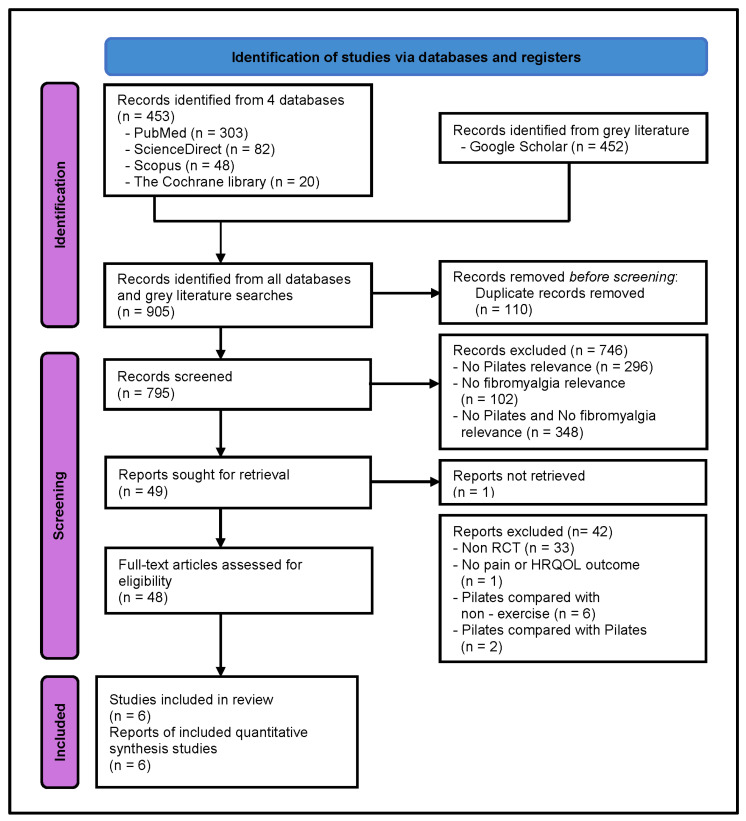
PRISMA flow diagram of selected studies.

A PRISMA flow diagram documented the study selection process and is presented in Figure 1. An initial database search yielded 905 studies. After 110 removing duplicates, 795 studies remained. Screening titles and abstracts led to the exclusion of 746 studies due to irrelevance to Pilates, fibromyalgia, or both. Access to one study was not possible, as an email request for the research received no response. After screening the full texts of 48 articles, 42 were excluded for not meeting the eligibility criteria. A detailed list of the excluded articles, along with the main reason for exclusion, is provided in Supplementary Table S1. In summary, six studies were included, involving a total of 265 patients. These RCTs were conducted in five Western countries [21,22,23,24,25] and one Asian country [26]. The number of included patients ranged from 20 to 97 patients [21,22,23,24,25,26]. Five studies assessed both pain and HRQOL outcomes [21,22,23,24,25], while one focused solely on pain [26]. No study reported adverse effects of Pilates training [21,22,23,24,25,26].

All included studies involved adult FMPs diagnosed according to the 1990 or 2010 ACR criteria (mean age, 45.5 to 56.28 years) [21,22,23,24,25,26]. Three studies included female participants only [21,22,25], while the others included both genders [23,24,26]. Five studies reported mean baseline FIQ scores ranging from 58.29 to 80.80 [21,22,23,24,25] (scores < 70 indicate moderate HRQOL, scores ≥ 70 indicate severe HRQOL) [27]. One study did not report baseline FIQ scores [26]. Regarding baseline pain, five studies reported mean baseline VAS scores between 6.1 and 8.88 [21,22,23,25,26] (scores ≤ 3.4 indicate mild pain, 3.5–7.4 indicate moderate pain, ≥7.5 indicate severe pain) [28].

The interventions consisted of mat Pilates [22,23] or mat Pilates with equipment (resistant band, Swiss ball, Cadillac, Reformer, ladder barrel) [21,24,25,26] compared to mind–body exercises (yoga or home exercise relaxation/stretching) [25,26], usual care [22,23], or aerobic exercises [21,24]. The studies had a duration of 50 to 60 min per session [22,23,24,25,26], with Pilates practiced two to six times per week for four to twelve weeks [21,22,23,24,25,26], as presented in Table 1 and Table 2.

### 3.2. Quality of Included Studies

All six studies underwent methodological appraisal using the PEDro scale [15,16], achieving scores ranging from 4 to 9, indicating moderate to excellent quality [21,22,23,24,25,26]. Four studies attained a score of 6 or higher [21,23,24,25], signifying rigorous research design, while the remaining were classified as having moderate methodological quality [22,26]. All studies met the predetermined inclusion criteria, ensuring adequate external validity [21,22,23,24,25,26]. Random allocation was employed in all studies; however, participant blinding was not feasible due to the nature of the exercise interventions. Three studies adopted an intention-to-treat analysis [21,24,26]. Effect sizes were reported as mean differences (MDs) with standard deviations (SDs) for all outcomes [21,22,23,24,25,26], which represented the point estimates and availability (Supplement Table S2).

### 3.3. Pain Outcomes

#### 3.3.1. Main Analysis

**Figure 2:**
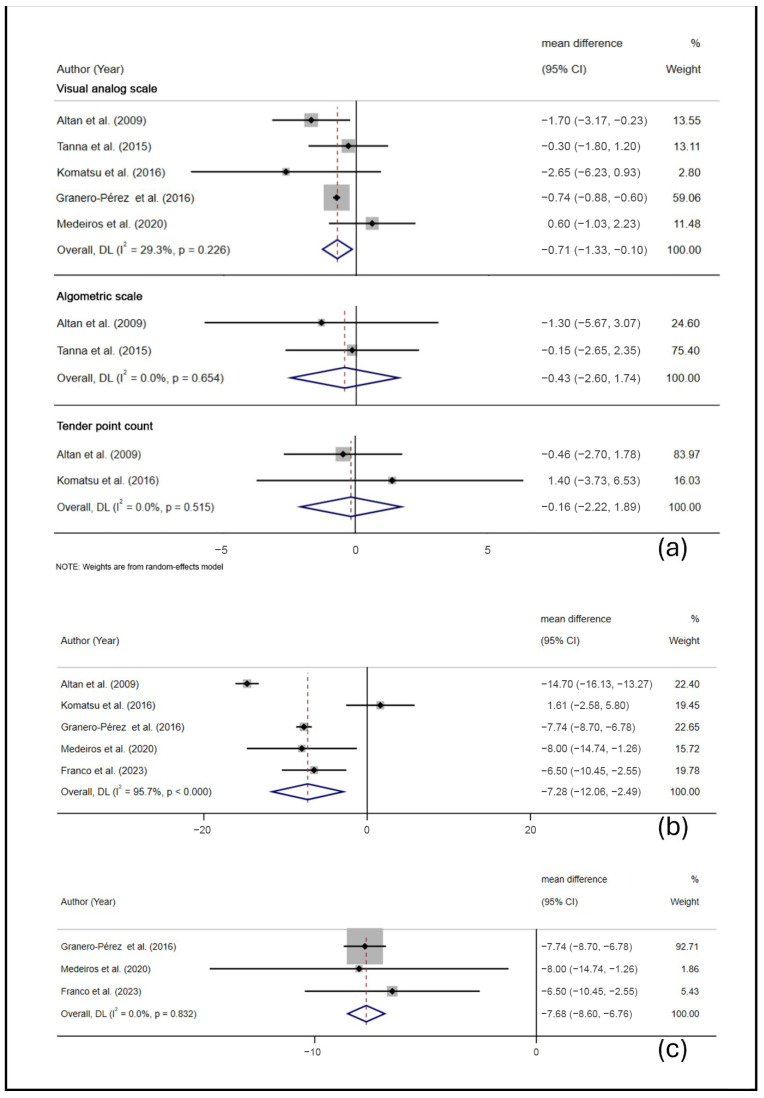
A forest plot of meta-analyses (**a**) of the effects of Pilates on pain measured by the VAS, AS, and TPC in FMPs [21,22,23,25,26]; (**b**) of the effects of Pilates on HRQOL measured by the FIQ in FMPs [21,22,23,24,25]; and (**c**) of sensitivity analyses of the effects of Pilates on HRQOL measured by the FIQ in FMPs [21,23,24].

The treatment effects of Pilates on pain from each individual study are reported in Table 3. A meta-analysis revealed the mixed effects of Pilates on pain. A pooled analysis of VAS scores from five RCTs showed significant pain reduction (MD, −0.71, 95% CI, −1.33 to −0.10, *p* = 0.023, *I*^2^ = 29.3%), while AS and TPC outcomes from two RCTs showed no significant differences (Figure 2a).

#### 3.3.2. Subgroup Analysis

Types of control: Significant pain reduction was found when Pilates was compared to usual care in two RCTs (MD, −0.82, 95% CI, −1.59 to −0.06, *p* = 0.035, *I*^2^ = 8.4%) but not against aerobic or mind–body exercise (Supplement Table S3).

Types of Pilates: Mat-based Pilates significantly reduced pain (MD, −0.82, 95% CI, −1.59 to −0.06, *p* = 0.035, *I*^2^ = 8.4%) compared to usual care, while mat with equipment Pilates showed no effect (MD, −0.50, 95%CI −1.81 to 0.81; *p* = 0.452, *I*^2^ = 54.2%) (Supplement Table S3).

Age: Pilates showed no significant effect for patients under 50 years but reduced pain significantly for those aged 50+ in one RCT (MD, −0.74, 95% CI, −0.88 to −0.60, *p* < 0.001) (Supplement Table S3).

### 3.4. HRQOL Outcome

#### 3.4.1. Main Analysis

The treatment effects of Pilates on HRQOL from each individual study are reported in Table 3. From our meta-analysis, Pilates significantly improved HRQOL (FIQ scores) in five studies (MD, −7.28, 95% CI, −12.06 to −2.49, *p* = 0.003, *I*^2^ = 95.7%) (Figure 2b). Sensitivity analysis using ACR 2010 criteria confirmed significant improvements with negligible heterogeneity (MD, −7.68, 95% CI, −8.60 to −6.76, *p* < 0.001, *I*^2^ = 0.0%) (Figure 2c).

#### 3.4.2. Subgroup Analysis

- Types of control: Pilates significantly reduced FIQ scores compared to mind–body (MD, −14.07, 95% CI, −16.13 to −13.27, *p* < 0.001) and aerobic exercise (MD, −6.88, 95% CI, −10.29 to −3.48, *p* < 0.001, *I*^2^ = 0.0%) but not compared to usual care (Supplement Table S3).
- Types of Pilates: Combined mat and equipment Pilates significantly improved FIQ scores (MD, −10.08, 95% CI, −16.39 to −3.76, p = 0.002, *I*^2^ = 88.5%). Mat-based Pilates alone showed no significant improvement (Supplement Table S3).
- Age: No significant FIQ score improvement for patients under 50 years was found, but significant improvement was observed for patients aged 50 or above (MD, −7.67, 95% CI, −8.60 to −6.74, p < 0.001, *I*^2^ = 0.0%) (Supplement Table S3).

## 4. Discussion

This meta-analysis comprehensively examined the effects of Pilates on pain and HRQOL in patients with fibromyalgia. Our findings suggest that Pilates may be a promising non-pharmacological intervention for managing fibromyalgia symptoms.

Our analysis revealed evidence of pain reduction, particularly as indicated by VAS scores. These findings align with previous research demonstrating Pilates’ effectiveness in reducing chronic low back pain [29]. However, inconsistencies in AS and TPC results may be attributed to differences in pain measurement tools and methodologies across studies [30]. The positive effects of Pilates on pain reduction in FMPs are consistent with broader research on exercise interventions for chronic pain conditions [29,31,32]. A systematic review and meta-analysis found that various types of exercise, including aerobic exercise and strength training, can improve pain outcomes in FMPs [8]. Our findings suggest that Pilates may be a valuable addition to this study of beneficial exercises.

Regarding HRQOL outcomes, both pooled-effect and sensitivity analyses demonstrated positive outcomes for HRQOL in FMPs who engaged in Pilates compared to control groups [21,22,23,24,25]. Although the pooled analysis showed high data variability, which may be due to differences in types of Pilates, types of control groups, gender, age, and diagnostic criteria for fibromyalgia [21,22,23,24,25], a sensitivity analysis was conducted by grouping studies diagnosed using the ACR 2010 criteria [21,23,24]. This approach significantly reduced data variability, and the results remained stable. These findings are consistent with numerous studies that have shown Pilates to enhance the quality of life in various populations, including the elderly, pregnant women, menopausal women, and patients with chronic low back pain [29,32,33,34]. The improvement in HRQOL is particularly significant given the multifaceted impact of fibromyalgia on patients’ daily lives and overall well-being [3].

Our subgroup analyses revealed several important insights, considering the Pilates modality. Mat-only Pilates was particularly beneficial for pain reduction, while a combination of mat and equipment Pilates showed benefits for HRQOL. This difference may be attributed to the nature of the exercises. Mat Pilates, which uses body weight as resistance, may be gentler and more suitable for pain management [10]. Equipment Pilates, on the other hand, allows for training specific muscles and deeper muscle layers, potentially leading to improved physical function and, consequently, better HRQOL [33]. When comparing Pilates to other forms of exercise or usual care, Pilates was found to be more effective in relieving pain than usual care [22,23] and superior to mind–body exercises and aerobic exercise in improving HRQOL [21,24,25]. These findings underscore the context-dependent efficacy of different exercise interventions, as noted in previous research [8]. Moreover, the subgroup analyses showed that people with fibromyalgia aged 50 and over experienced more significant improvements in both pain reduction and HRQOL [23,24]. This suggests that Pilates may be particularly beneficial for older adults with fibromyalgia, a finding that aligns with research on the benefits of Pilates for the elderly population [34]. As people age, they often experience lower hormone levels, posture instability, reduced muscle mass, and muscle pain [34]. The beneficial effects of Pilates on fibromyalgia symptoms in older adults may be attributed to several mechanisms. Pilates emphasizes core strength, flexibility, and body awareness, which can contribute to improved posture, reduced musculoskeletal pain, and increased muscle mass [9,25]. Additionally, the mind–body component of Pilates may aid in managing the psychological aspects of chronic pain, such as anxiety and depression, which are common comorbidities in fibromyalgia [3,34].

### 4.1. Clinical Implications

Our findings suggest that healthcare providers might consider recommending Pilates as a non-pharmacological option for fibromyalgia management, particularly for patients aged 50 years and above. The potential benefits of Pilates, combined with its low risk of adverse effects, make it an attractive complementary therapy to standard fibromyalgia treatments. This is especially relevant for elderly individuals, who are at higher risk of musculoskeletal issues and falls, which can contribute to broader societal challenges. However, it is crucial to note that Pilates should be tailored to individual patients’ needs and capabilities. The type of Pilates (mat vs. equipment), intensity, and duration should be carefully considered based on the patient’s condition and treatment goals. Specifically, mat Pilates appears to be more suitable for patients focusing on pain reduction, while mat Pilates combined with equipment may be better suited for those aiming to improve HRQOL.

### 4.2. Limitations and Future Directions

This meta-analysis reveals geographical bias in the included randomized controlled trials (RCTs), with most studies conducted in South America and Europe but only one from Asia. This limits the generalizability of the findings to diverse populations, as ethnicity and genetics can affect muscle development and body flexibility, leading to different exercise outcomes among various ethnic groups in studies. Additionally, the high proportion of female participants restricts conclusions regarding Pilates’ benefits for male FMPs, given potential gender-specific differences in symptoms and treatment response. The small sample sizes, heterogeneity of study populations, and limited number of studies highlight the need for further research. Publication bias may also distort the overall understanding, but the small number of included studies makes it difficult to assess this bias fully. Moreover, the short follow-up periods in most studies further limit the evaluation of Pilates’ long-term effects on pain and HRQOL in fibromyalgia patients.

Future studies should focus on larger, multi-center RCTs with more male and diverse participants to enhance the generalizability and statistical power of the findings. Long-term follow-up studies are also needed to determine the optimal duration and frequency of Pilates exercises for maximum benefit. Furthermore, research should explore the combination of Pilates with other treatments, such as pharmacological interventions, or other non-pharmacological approaches including cognitive–behavioral therapy or education programs. For instance, programs like “Amigos de Fibro”, which focus on education, empowerment, providing support networks, and integrating care for fibromyalgia patients, could complement Pilates and lead to further improvements in patient outcomes [35,36]. These combined approaches could contribute to the development of evidence-based guidelines for the comprehensive management of fibromyalgia.

## 5. Conclusions

This systematic review and meta-analysis provide important evidence supporting the use of Pilates as an effective intervention for reducing pain and HRQOL among patients with fibromyalgia. While these findings are promising, healthcare providers should consider specific patient demographics and controlled conditions. The limitations of the current evidence base necessitate cautious interpretation. Further high-quality research is needed to solidify the role of Pilates in the comprehensive management of fibromyalgia and to guide clinical recommendations more precisely.

## Figures and Tables

**Table 1:** Characteristics of included studies.

Author/Year/ Country	Intervention	N	ACR	Sex	Age (Mean)	FIQ Baseline (Mean)	VAS Baseline (Mean)	Outcome Measurement
Altan et al., 2009 [25] Turkey	Mat Pilates with resistant band and ball (time: 60 min/session, 3 sessions/week × 12 weeks)	25	1990	female	48.2	80.80	6.10	Pain: AS/TPC/VAS HRQOL: FIQ/NHP
	Home exercise relaxation/ stretching group (time: 60 min/session, 3 sessions/week × 12 weeks)	24	1990	female	50	80.10	6.30	
Tanna et al., 2015 [26] India	Stott Pilates (time: 60 min/sessions, 6 sessions/week × 4 weeks)	10	1990	both sex	not told	not told	7.50	Pain: AS/VAS/TPI HRQOL: -
	Yoga (time: 60 min/sessions, 6 sessions/week × 4 weeks)	10	1990	both sex	not told	not told	6.90	
Komatsu et al., 2016 [22] Brazil	Mat Pilates (time: 60 min/sessions, 2 sessions/week × 8 weeks)	13	1990	female	47.85	68.85	8.88	Pain: TPC/VAS HRQOL: FIQ
	Usual treatment without any exercise	7	1990	female	53.29	58.29	6.86	
Granero-Pérez et al., 2016 [23] Spain	Mat Pilates (time: 60 min/sessions, 6 sessions/week × 12 weeks)	18	2010	both sex	56.28	62.92	7.89	Pain: VAS HRQOL: FIQ
	Usual treatment without any exercise	19	2010	both sex	53.62	65.13	7.74	
Medeiros et al., 2020 [21] Brazil	Mat Pilates with Swiss ball (time: 50 min/sessions, 2 sessions/week × 12 weeks)	21	2010	female	45.5	68	7.89	Pain: VAS HRQOL: FIQ/SF-36
	Aquatic aerobic exercises (time: 40 min/sessions, 2 sessions/week × 12 weeks)	21	2010	female	50.7	67	7.50	
Franco et al., 2023 [24] Brazil	Mat Pilates and equipment-based Pilates (Cadillac, Reformer, ladder barrel, step chair) (time: 60 min/sessions, 2 sessions/week × 8 weeks)	48	2010	both sex	51.4	70.3	not told	Pain: NRS HRQOL: FIQ/EQ-5D
	Aerobic exercises (time: 60 min/sessions, 2 sessions/week × 8 weeks)	49	2010	both sex	48.5	68.3	not told	

Abbreviations: ACR = American College of Rheumatology; AS = algometric scale; EQ-5D = EuroQol, five dimensions; FIQ = Fibromyalgia Impact Questionnaire; NHP = Nottingham Health Profile; NRS = numeric rating scale; SF 36 = short form-36 health; TPC = tender point count; VAS = visual analog scale.

**Table 2:** Pilates training characteristics of included studies.

Author, Year	Type of Pilates	With Warm up and Cool down	With Increasing Intensity	Length of Time (Minutes/ Session)	Frequency (Sessions/ Week)	Duration of Exercise (Weeks)
Altan et al., 2009 [25]	Mat Pilates with resistant band and ball	-	-	60	3	12
Tanna et al., 2015 * [26]	Stott Pilates	√	-	60	6	4
Komatsu et al., 2016 [22]	Mat Pilates	-	√	60	2	8
Granero-Pérez et al., 2016 [23]	Mat Pilates	√	-	60	2	12
Medeiros et al., 2020 [21]	Mat Pilates with Swiss ball	-	√	50	2	12
Franco et al., 2023 ** [24]	Mat Pilates and equipment-based Pilates (Cadillac, Reformer, ladder barrel, step chair)	√	√	60	2	8

* = no FIQ measurement; ** = no VAS measurement.

**Table 3:** The mean difference and standard deviations of each included study used in the meta-analysis.

Author, Year	Intervention	Control	Measurement	Outcome			
				Intervention		Control	
				MD	SD	MD	SD
Altan et al., 2009 [25]	Mat Pilates with resistant band and ball	home exercise relaxation/ stretching group	VAS	−17.30	2.40	−2.60	2.70
			AS	10.20	1.70	11.50	10.80
			TPC	−3.56	3.60	−3.10	4.38
			FIQ	−17.30	2.40	−2.60	2.70
Tanna et al., 2015 [26]	Stott Pilates	Yoga	VAS	−2.90	1.87	−2.60	1.54
			AS	4.84	3.95	4.99	0.80
Komatsu et al., 2016 [22]	Mat Pilates	Usual treatment	VAS	−2.80	3.12	−0.15	4.25
			TPC	−1.31	6.00	−2.71	4.37
			FIQ	−13.89	7.55	−15.5	1.15
Granero-Pérez et al., 2016 [23]	Mat Pilates	Usual treatment	VAS	1.22	0.16	−0.48	0.26
			FIQ	−13.89	7.55	−15.5	1.15
Medeiros et al., 2020 [21]	Mat Pilates with Swiss ball	Aquatic aerobic exercise	VAS	−1.30	1.69	−1.90	3.41
			FIQ	−17.00	15.6	−9.00	2.26
Franco et al., 2023 [24]	Mat Pilates and Equipment based Pilates	aerobic exercise	NRS	3.70	2.70	4.90	2.80
			FIQ	−31.3	9.2	−24.8	10.6

Abbreviations: AS = algometric scale; FIQ = Fibromyalgia Impact Questionnaire; MD = mean difference; NRS = numeric rating scale; SD = standard deviation; TPC = tender point count; VAS = visual analog scale.

## Data Availability

Data available on reasonable request to the corresponding author.

## Supplementary Materials

The following supporting information can be downloaded at: https://www.mdpi.com/article/10.3390/jcm13237447/s1, Table S1: Reason of exclusion; Table S2: Quality assessment of 6 included studies in systematic review and meta-analysis, evaluated by PEDro scale; Table S3: Effect of Pilates on pain and HRQOL outcomes in fibromyalgia patients (subgroup analyses and sensitivity analyses).

## References

[1] Bidonde J., Busch A.J., Schachter C.L., Webber S.C., Musselman K.E., Overend T.J., Góes S.M., Dal Bello-Haas V., Boden C., Cochrane Musculoskeletal Group (1996). Mixed exercise training for adults with fibromyalgia. Cochrane Database Syst. Rev..

[2] Shuster J., McCormack J., Riddell R.P., Toplak M.E. (2009). Understanding the psychosocial profile of women with fibromyalgia syndrome. Pain Res. Manag..

[3] Queiroz L.P. (2013). Worldwide epidemiology of fibromyalgia. Curr. Pain Headache Rep..

[4] Fitzcharles M.-A., Ste-Marie P.A., Goldenberg D.L., Pereira J.X., Abbey S., Choinière M., Ko G., Moulin D.E., Panopalis P., Proulx J. (2013). 2012 Canadian guidelines for the diagnosis and management of fibromyalgia syndrome: Executive summary. Pain Res. Manag..

[5] Lee L.K., Ebata N., Hlavacek P., DiBonaventura M., Cappelleri J.C., Sadosky A. (2016). Humanistic and economic burden of fibromyalgia in Japan. J. Pain Res..

[6] Plazier M., Ost J., Stassijns G., De Ridder D., Vanneste S. (2015). Pain characteristics in fibromyalgia: Understanding the multiple dimensions of pain. Clin. Rheumatol..

[7] Macfarlane G.J., Kronisch C., Dean L., Atzeni F., Häuser W., Fluß E., Choy E., Kosek E., Amris K., Branco J. (2017). EULAR revised recommendations for the management of fibromyalgia. Ann. Rheum. Dis..

[8] Sosa-Reina M.D., Nunez-Nagy S., Gallego-Izquierdo T., Pecos-Martín D., Monserrat J., Álvarez-Mon M. (2017). Effectiveness of therapeutic exercise in fibromyalgia syndrome: A systematic review and meta-analysis of randomized clinical trials. BioMed Res. Int..

[9] Penelope L. (2002). Updating the principles of the Pilates method—Part 2. J. Bodyw. Mov. Ther..

[10] Caglayan B.C., Calik B.B., Kabul E.G., Karasu U. (2023). Investigation of effectiveness of reformer pilates in individuals with fibromyalgia: A randomized controlled trial. Reumatol. Clin..

[11] Ekici G., Unal E., Akbayrak T., Vardar-Yagli N., Yakut Y., Karabulut E. (2017). Effects of active/passive interventions on pain, anxiety, and quality of life in women with fibromyalgia: Randomized controlled pilot trial. Women Health.

[12] Page M.J., McKenzie J.E., Bossuyt P.M., Boutron I., Hoffmann T.C., Mulrow C.D., Shamseer L., Tetzlaff J.M., Akl E.A., Brennan S.E. (2021). The PRISMA 2020 statement: An updated guideline for reporting systematic reviews. BMJ.

[13] Higgins J.P., Green S. (2008). Cochrane Handbook for Systematic Reviews of Interventions.

[14] Li L., Asemota I., Liu B., Gomez-Valencia J., Lin L., Arif A.W., Siddiqi T.J., Usman M.S. (2022). AMSTAR 2 appraisal of systematic reviews and meta-analyses in the field of heart failure from high-impact journals. Syst. Rev..

[15] Maher C.G., Sherrington C., Herbert R.D., Moseley A.M., Elkins M. (2003). Reliability of the PEDro scale for rating quality of randomized controlled trials. Phys. Ther..

[16] Cashin A.G. (2020). Clinimetrics: Physiotherapy evidence database (PEDro) scale. J. Physiother..

[17] Reitsma J., Rutjes A., Whiting P., Vlassov V., Leeflang M., Deeks J. (2009). Assessing methodological quality. Cochrane Handbook for Systematic Reviews of Diagnostic Test Accuracy Version 1.0.

[18] Lee D.K. (2016). Alternatives to P value: Confidence interval and effect size. Korean J. Anesthesiol..

[19] DerSimonian R., Laird N. (1986). Meta-analysis in clinical trials. Control. Clin. Trials.

[20] Higgins J.P., Thompson S.G., Deeks J.J., Altman D.G. (2003). Measuring inconsistency in meta-analyses. BMJ.

[21] Medeiros S.A.d., Silva H.J.d.A., Nascimento R.M.d., Maia J.B.d.S., Lins C.A.d.A., Souza M.C.d. (2020). Mat Pilates is as effective as aquatic aerobic exercise in treating women with fibromyalgia: A clinical, randomized and blind trial. Adv. Rheumatol..

[22] Komatsu M., Avila M.A., Colombo M.M., Gramani-Say K., Driusso P. (2016). Pilates training improves pain and quality of life of women with fibromyalgia syndrome. Rev. Dor.

[23] Granero-Pérez M. (2017). Efectos Inmediatos de un Programa de Ejercicios de Pilates Sobre el Equilibrio y la Calidad de vida de Mujeres con Fibromialgia. https://crea.ujaen.es/handle/10953.1/4698.

[24] Franco K.F.M., Miyamoto G.C., Franco Y.R.d.S., Salvador E.M.E.S., do Nascimento B.C.B., Menten L.A., Cabral C.M.N. (2023). Is Pilates more effective and cost-effective than aerobic exercise in the treatment of patients with fibromyalgia syndrome? A randomized controlled trial with economic evaluation. Eur. J. Pain.

[25] Altan L., Korkmaz N., Bingol Ü., Gunay B. (2009). Effect of pilates training on people with fibromyalgia syndrome: A pilot study. Arch. Phys. Med. Rehabil..

[26] Tanna A., Basu S., Anadkat K. (2015). Effects of stott’s pilates versus yogic exercise in fibrom yalgia patients: A pilot study. Int. J. Physiother. Res..

[27] Bennett R. (2005). The Fibromyalgia Impact Questionnaire (FIQ): A review of its development, current version, operating characteristics and uses. Clin. Exp. Rheumatol..

[28] Boonstra A.M., Schiphorst Preuper H.R., Balk G.A., Stewart R.E. (2014). Cut-off points for mild, moderate, and severe pain on the visual analogue scale for pain in patients with chronic musculoskeletal pain. Pain.

[29] Fernández-Rodríguez R., Álvarez-Bueno C., Cavero-Redondo I., Torres-Costoso A., Pozuelo-Carrascosa D.P., Reina-Gutiérrez S., Pascual-Morena C., Martínez-Vizcaíno V. (2022). Best exercise options for reducing pain and disability in adults with chronic low back pain: Pilates, strength, core-based, and mind-body. a network meta-analysis. J. Orthop. Sports Phys. Ther..

[30] Smith A.K., Togeiro S.M., Tufik S., Roizenblatt S. (2009). Disturbed sleep and musculoskeletal pain in the bed partner of patients with obstructive sleep apnea. Sleep Med..

[31] Taşpınar G., Angın E., Oksüz S. (2023). The effects of Pilates on pain, functionality, quality of life, flexibility and endurance in lumbar disc herniation. J. Comp. Eff. Res..

[32] Sonmezer E., Özköslü M.A., Yosmaoğlu H.B. (2021). The effects of clinical pilates exercises on functional disability, pain, quality of life and lumbopelvic stabilization in pregnant women with low back pain: A randomized controlled study. J. Back Musculoskelet. Rehabil..

[33] Lee C.-W., Hyun J., Kim S.G. (2014). Influence of pilates mat and apparatus exercises on pain and balance of businesswomen with chronic low back pain. J. Phys. Ther. Sci..

[34] Narici M.V., Maffulli N. (2010). Sarcopenia: Characteristics, mechanisms and functional significance. Br. Med. Bull..

[35] Antunes M.D., da Rocha Loures F.C.N., de Souza I.M.B., Cruz A.T., de Oliveira Januário P., Pinheiro M., Schmitt A.C.B., Frutos-Bernal E., Martín-Nogueras A.M., Marques A.P. (2023). A web-based educational therapy intervention associated with physical exercise to promote health in fibromyalgia in Brazil: The Amigos De Fibro (Fibro Friends) study protocol. Trials.

[36] Antunes M.D., Schmitt A.C.B., Marques A.P. (2022). Amigos de Fibro (Fibro Friends): Development of an educational program for the health promotion of fibromyalgia patients. Prim. Health Care Res. Dev..

