# Supplementary material for: Effect of Pilates on Pain and Health-Related Quality of Life in Fibromyalgia Patients: A Systematic Review and Meta-Analysis
Source: J Clin Med. 2024 Dec 6;13(23):7447. doi: 10.3390/jcm13237447 (PMC11642570; doi:10.3390/jcm13237447)
Supplement: Supplementary file 1 [file jcm-13-07447-s001.zip › jcm-3336192-supplementary.pdf]

Supplementary

Effect of Pilates on Pain and Health-Related Quality of Life in Fibromyalgia Patients: A Systematic Review and Meta-Analysis

Chalisa Nithuthorn [1], Natapohn Chaipichit [2], Thammasorn Jeeraaumponwat [3], Mart Maiprasert [1,*] and Piyameth Dilokthornsakul [2,*]

[1] Department of Anti-Aging and Regenerative Medicine College of Integrative Medicine, Dhurakij Pundit University, Laksi, Bangkok 10210, Thailand
[2] Center for Medical and Health Technology Assessment (CM-HTA), Department of Pharmaceutical Care, Faculty of Pharmacy, Chiang Mai University, Chiang Mai 50200, Thailand
[3] Department of Social Medicine, Khon Kaen Hospital, Srichan Rd, Nai Mueang, Mueang, Khon Kaen 40000, Thailand
* Correspondence: mart.mai@dpu.ac.th (M.M.); piyameth.dilok@cmu.ac.th (P.D.); Tel.: +66-86699-3900 (M.M.); +66-86735-4746 (P.D.)

Table S1. A list of excluded articles.

NO.	Author, Year	Title	Reason of exclusion
1	Almagro et al. 2023	Optimal dose and type of exercise to reduce pain, anxiety and increase quality of life in patients with fibromyalgia. A systematic review with meta-analysis	Not RCT
2	Antunes et al. 2022	The role of physiotherapy in fibromyalgia: Current and future perspectives	Not RCT
3	Araya-Quintanilla et al. 2022	Effectiveness of multicomponent treatment in patients with fibromyalgia: protocol for a systematic review and meta-analysis	Not RCT
4	Ardila et al. 2020	Effectiveness of acupuncture vs. core stability training in balance and functional capacity of women with fibromyalgia: a randomized controlled trial	Pilates vs. non-exercise
5	Bidonde et al. 2019	Mixed exercise training for adults with fibromyalgia	Not RCT
6	Bidonde et al. 2023	Effectiveness of non-pharmacological interventions for fibromyalgia and quality of review methods: an overview of Cochrane Reviews	Not RCT
7	Bush et al. 2011	Exercise Therapy for Fibromyalgia	Not RCT
8	Caglayan et al. 2006	FRI0704-HPR The effects of clinical Pilates training in patients with fibromyalgia: a randomized controlled trial	Pilates vs. Pilates
**9**	Caglayan et al. 2022	Investigation of effectiveness of reformer Pilates in individuals with fibromyalgia: A randomized controlled trial	Pilates vs. Pilates
10	Calvo et al. 2023	Are physical therapy interventions effective in improving sleep in people with chronic pain? A systematic review and multivariate meta-analysis	Not RCT
11	Celeney et al. 2017	A comparison of the effects of exercises plus connective tissue massage to exercises alone in women with fibromyalgia syndrome: a randomized controlled trial	Pilates vs. non-exercise

NO.	Author, Year	Title	Reason of exclusion
12	Cordeiro et al. 2020	Influence of the Pilates method on quality of life and pain of individuals with fibromyalgia: integrative review	Not RCT
13	Courtois et al. 2015	Effectiveness of body awareness interventions in fibromyalgia and chronic fatigue syndrome: A systematic review and meta-analysis	Not RCT
14	Cruz et al, 2023	Evaluation of the Treatment of Patients with Fibromyalgia at the Clinical School of Physiotherapy of the University of Gurupi	Not RCT
15	Dragoi et al. 2020	The effect of different non-pharmacological therapies on quality of life in fibromyalgia patients	Not RCT
16	Ekici et al. 2008	**Effects of Pilates exercises and connective tissue manipulation on pain and depression in females with fibromyalgia: a randomized controlled trial**	Pilates vs. non-exercise
17	Ekici et al. 2016	Effects of active/passive interventions on pain, anxiety, and quality of life in women with fibromyalgia: randomized controlled pilot trial	Pilates vs. non-exercise
18	Ferraz et al. 2023	Effect of the pilates method on pain and quality of life in pregnancy: A systematic review and meta-analysis	Not RCT
19	Frietas et al. 2020	Effects of the pilates method on kinesiophobia associated with chronic non-specific low back pain: Systematic review and meta-analysis	Not RCT
20	Garcia et al. 2020	Effectiveness of active therapy-based training to improve the balance in patients with fibromyalgia: A Systematic Review with Meta-Analysis	Not RCT
21	Geneen et al. 2017	Physical activity and exercise for chronic pain in adults: an overview of Cochrane Reviews	Not RCT
22	Gulsen et al. 2022	Effect of fully immersive virtual reality treatment combined with exercise in fibromyalgia patients: a randomized controlled trial	Pilates vs. non-exercise
23	Jesus et al. 2022	The use of Pilates for pain control in patients with fibromyalgia	Not RCT
24	Jones et al. 2009	Exercise Interventions in Fibromyalgia: Clinical Applications from the Evidence	Not RCT

NO.	Author, Year	Title	Reason of exclusion
25	Keskin et al. 2020	AB0954 Is connective tissue massage effective in individuals with fibromyalgia?	Pilates vs. non-exercise
26	Kim et al. 2019	Flexibility exercise training for adults with fibromyalgia	Not RCT
27	Korkmaz et al. 2010	Effects of Pilates Exercises on the Social Physical Concern of Patients with Fibromyalgia Syndrome: A Pilot Study	Not RCT
28	Laura et al. 2022	Do patients with fibromyalgia have body image and tactile acuity distortion?	no pain or HRQOL outcome
29	Manojlovic et al. 2023	The effectiveness of aerobic exercise for pain management in patients with fibromyalgia	Not RCT
30	Martínez-Rodríguez et al. 2018	Effects of lacto-vegetarian diet and stabilization core exercises on body composition and pain in women with fibromyalgia: randomized controlled trial	Pilates vs. non-exercise
31	Melissa et al. 2015	Pilates Method for Women's Health: Systematic Review of Randomized Controlled Trials	Not RCT
32	Mendoza et al. 2016	Benefits of physical exercise in postmenopausal women	Not RCT
33	Miranda et al. 2018	Pilates in noncommunicable diseases: A systematic review of its effects	Not RCT
34	Mist et al. 2013	Complementary and alternative exercise for fibromyalgia: a meta-analysis	Not RCT
35	Moreira et al. 2021	Pool-based exercise for amelioration of pain in adults with fibromyalgia syndrome: A systematic review and meta-analysis	Not RCT
36	Offenbäche et al. 2007	Are the contents of treatment outcomes in fibromyalgia trials represented in the International Classification Of Functioning, Disability, and Health?	Not RCT
37	Parveen et al. 2023	Effects of Pilates on health and well-being of women: a systematic review	Not RCT

NO.	Author, Year	Title	Reason of exclusion
38	Plata et al. 2023	Effectiveness of aquatic therapy on sleep in persons with fibromyalgia. A meta-analysis	Not RCT
39	Silva et al. 2019	Mat Pilates and aquatic aerobic exercises for women with fibromyalgia: a protocol for a randomised controlled blind study	Not RCT
40	Sousa et al. 2023	Effects of Combined Training Programs in Individuals with Fibromyalgia: A Systematic Review	Not RCT
41	Tunks et al. 1995	The reliability of examination for tenderness in patients with myofascial pain, chronic fibromyalgia and controls	Not RCT
42	Vasileios et al. 2022	Managing fibromyalgia with complementary and alternative medical exercise: a systematic review and meta-analysis of clinical trials	Not RCT

Table S2. Quality assessment of six included studies in systematic review and meta-analysis, evaluated by PEDro scale.

**Items**	**Altan et al. 2009 [1]**	**Tanna et al. 2015 [2]**	**Komatsu et al. 2016 [3]**	**Granero-Pérez et al. 2016 [4]**	**Medeiros et al. 2020 [5]**	**Franco et al. 2023 [6]**
Eligibility criteria	1	1	1	1	1	1
Random allocation	1	1	1	1	1	1
Concealed allocation	0	0	0	0	1	1
Baseline comparability	1	0	1	1	1	1
Blind subjects	0	0	0	0	0	0
Blind therapist	0	0	0	0	1	0
Blind assessors	1	0	1	1	1	1
Measures ≥ 85% of the sample	1	1	0	1	1	1
Intention to treat	0	1	0	0	1	1
Between group comparison	1	1	0	1	1	1
Point estimates and availability	1	1	1	1	1	1
**PEDro scale**	**6**	**5**	**4**	**6**	**9**	**8**

Table S3. Effect of Pilates on pain and HRQOL outcomes in fibromyalgia patients (subgroup analyses and sensitivity analyses).

Condition	Subgroup or Sensitivity analysis	N	MD (95%CI); *p-value*	I^2(%); *p-value* (heterogeneity)	Included studies
**Pain outcome**					
1. Subgroup by type of control	Pilates vs. mind–body exercise	69	-1.01 (-2.38, 0.36); *p*=0.150	41.4; *p*=0.191	Altan et al. [1], Tanna et al. [2]
	Pilates vs. usual care	57	-0.82 (-1.59, -0.06); *p*=0.035	8.4; *p*=0.296	Komatsu et al. [3], Granero-Pérez et al. [4]
	Pilates vs. aerobic exercise	42	0.60 (-1.03, 2.23); *p*=0.471	-	Medeiros et al. [5]
2. Subgroup by type of Pilates exercise	Mat Pilates and equipment Pilates	111	-0.50 (-1.81, 0.81); *p*=0.452	54.2; *p*=0.113	Altan et al. [1], Tanna et al. [2], Medeiros et al. [5]
	Mat Pilates	57	-0.82 (-1.59, -0.06); *p*=0.035	8.4; *p*=0.296	Komatsu et al. [3], Granero-Pérez et al. [4]
3. Subgroup by age	Age: less than 50 years old	111	-0.97 (-2.85, 0.92); *p*=0.314	62.2; *p*=0.071	Altan et al. [1], Komatsu et al. [3], Medeiros et al. [5]
	Age: less than 50 years old with sensitivity analysis	69	-1.84 (-3.20, -0.48); *p*=0.008	0.0; *p*=0.630	Altan et al. [1], Komatsu et al. [3]
	Age: more than or equal 50 years	37	-0.74 (-0.88, -0.60); *p*<0.001	-	Granero-Pérez et al. [4]
**HRQOL outcome**					
	Pool FIQ with sensitivity analysis	176	-7.68 (-8.60, -6.76); *p*<0.001	0.0; *p*=0.832	Granero-Pérez et al. [4], Medeiros et al. [5], Franco et al. [6]
1. Subgroup by type of control	Pilates vs. mind–body exercise	49	-14.70 (-16.13, -13.27); *p*<0.001	-	Altan et al. [1]
	Pilates vs. usual care	57	-3.30 (-12.45, 5.85); *p*=0.480	94.5; *p*<0.001	Komatsu et al. [3], Granero-Pérez et al. [4]
	Pilates vs. aerobic exercise	139	-6.88 (-10.29, -3.48); *p*<0.001	0.0; *p*=0.707	Medeiros et al. [5], Franco et al. [6]
2. Subgroup by type of Pilates exercise	Mat Pilates and equipment Pilates	188	-10.08 (-16.39, -3.76); *p*=0.002	88.5; *p*<0.001	Altan et al. [1], Medeiros et al. [5], Franco et al. [6]
	Mat Pilates and equipment Pilates with sensitivity analysis	139	-6.88 (-10.29, -3.48); *p*<0.001	0.0; *p*=0.707	Medeiros et al. [5], Franco et al. [6]
	Mat Pilates	57	-3.30 (-12.45, -5.85); *p*=0.480	94.5; *p*<0.001	Komatsu et al. [3], Granero-Pérez et al. [4]
3. Subgroup by age	Age: less than 50 years old	111	-7.12 (-18.55, 4.32); *p*=0.223	96.3; *p*<0.001	Altan et al. [1], Komatsu et al. [3], Medeiros et al. [5]
	Age: more than or equal 50 years	134	-7.67 (-8.60, -6.74); *p*<0.001	0.0; *p*=0.550	Granero-Pérez et al. [4], Franco et al. [6]

Abbreviations: ACR = American College of Rheumatology; AS = algometric scale; FIQ = Fibromyalgia Impact Questionnaire; MD = mean difference; I^2 = heterogeneity; p = p-value; TPC = tender point count; VAS = visual analog scale; 95% CI = 95% confidence interval.

References

1. Altan, L.; Korkmaz, N.; Bingol, Ü.; Gunay, B. Effect of pilates training on people with fibromyalgia syndrome: a pilot study. *Archives of physical medicine and rehabilitation* **2009**, *90*, 1983-1988.
2. Tanna, A.; Basu, S.; Anadkat, K. EFFECTS OF STOTT'S PILATES VERSUS YOGIC EXERCISE IN FIBROM YALGIA PATIENTS: A PILOT STUDY. *Int J Physiother Res* **2015**, *3*, 1250-1257.
3. Komatsu, M.; Avila, M.A.; Colombo, M.M.; Gramani-Say, K.; Driusso, P. Pilates training improves pain and quality of life of women with fibromyalgia syndrome. *Revista Dor* **2016**, *17*, 274-278.
4. Granero-Pérez, M. Efectos inmediatos de un programa de ejercicios de pilates sobre el equilibrio y la calidad de vida de mujeres con fibromialgia. **2017**.
5. Medeiros, S.A.d.; Silva, H.J.d.A.; Nascimento, R.M.d.; Maia, J.B.d.S.; Lins, C.A.d.A.; Souza, M.C.d. Mat Pilates is as effective as aquatic aerobic exercise in treating women with fibromyalgia: a clinical, randomized and blind trial. *Advances in Rheumatology* **2020**, *60*, 21.
6. Franco, K.F.M.; Miyamoto, G.C.; Franco, Y.R.d.S.; Salvador, E.M.E.S.; do Nascimento, B.C.B.; Menten, L.A.; Cabral, C.M.N. Is Pilates more effective and cost-effective than aerobic exercise in the treatment of patients with fibromyalgia syndrome? A randomized controlled trial with economic evaluation. *European Journal of Pain* **2023**, *27*, 54-71.
